# Skp2B Overexpression Alters a Prohibitin-p53 Axis and the Transcription of PAPP-A, the Protease of Insulin-Like Growth Factor Binding Protein 4

**DOI:** 10.1371/journal.pone.0022456

**Published:** 2011-08-04

**Authors:** Harish Chander, Max Halpern, Lois Resnick-Silverman, James J. Manfredi, Doris Germain

**Affiliations:** 1 Division of Hematology/Oncology, Department of Medicine, Mount Sinai School of Medicine, New York, New York, United States of America; 2 Department of Oncological Sciences, Mount Sinai School of Medicine, New York, New York, United States of America; The University of Hong Kong, China

## Abstract

**Background:**

We previously reported that the degradation of prohibitin by the SCF^Skp2B^ ubiquitin ligase results in a defect in the activity of p53. We also reported that MMTV-Skp2B transgenic mice develop mammary gland tumors that are characterized by an increased proteolytic cleavage of the insulin-like growth factor binding protein 4 (IGFBP-4), an inhibitor of IGF signaling. However, whether a link exists between a defect in p53 activity and proteolysis of IGFBP-4 was not established.

**Methods and Results:**

We analyzed the levels of pregnancy-associated plasma protein A (PAPP-A), the protease of IGFBP-4, in MMTV-Skp2B transgenic mice and found that PAPP-A levels are elevated. Further, we found a p53 binding site in intron 1 of the PAPP-A gene and that both wild type and mutant p53 bind to this site. However, binding of wild type p53 results in the transcriptional repression of PAPP-A, while binding of mutant p53 results in the transcriptional activation of PAPP-A. Since MMTV-Skp2B mice express wild type p53 and yet show elevated levels of PAPP-A, at first, these observations appeared contradictory. However, further analysis revealed that the defect in p53 activity in Skp2B overexpressing cells does not only abolish the activity of wild type of p53 but actually mimics that of mutant p53. Our results suggest that in absence of prohibitin, the half-life of p53 is increased and like mutant p53, the conformation of p53 is denatured.

**Conclusions:**

These observations revealed a novel function of prohibitin as a chaperone of p53. Further, they suggest that binding of denatured p53 in intron 1 causes an enhancer effect and increases the transcription of PAPP-A. Therefore, these findings indicate that the defect in p53 function and the increased proteolysis of IGFBP-4, we had observed, represent two components of the same pathway, which contributes to the oncogenic function of Skp2B.

## Introduction

F-box proteins act as the substrate recognition subunits of specific ubiquitin ligase complexes. Skp2 is one of the best characterized F-box proteins and has been implicated in the degradation of several key regulators of the cell cycle and checkpoint controls. Like other F-box proteins such as β-TRCP, Skp2 has been reported to have three alternative splice forms, Skp2 or Skp2A, Skp2B and Skp2-gamma, although Skp2-gamma remains uncharacterized. We previously reported that Skp2A and Skp2B are both overexpressed in breast cancers [1] and that Skp2B is distinct from Skp2A at several levels; Skp2B localizes to the cytoplasm rather than the nucleus, further, Skp2B is short lived compared to Skp2A and does not show a significant effect on Skp2A substrates [1].

In order to determine whether Skp2B does play a role in breast cancer, we established transgenic mice overexpressing Skp2B in the mammary gland under the control of the mouse mammary tumor virus (MMTV) promoter [2]. We found that MMTV-Skp2B mice develop a number of phenotypes including acceleration of the invasion of the fat pad during puberty, increased side branching, pregnancy-like phenotype in virgin females and mammary tumors [2].

We identified the repressor of the estrogen receptor activity (REA), as a Skp2B binding protein. Further, since REA heterozygote mice also display an accelerated fat pad invasion and accelerated proliferation of the mammary gland during pregnancy [3], these observations suggested that Skp2B may affect REA degradation. In agreement with this possibility, we reported that Skp2B does lead to the ubiquitin-dependent degradation of REA [2]. However, unlike REA heterozygote mice, MMTV-Skp2B mice develop mammary gland tumors suggesting that Skp2B has additional substrates.

We also reported that Skp2B promotes the degradation of prohibitin [4], a protein associated with multiple functions including a role as a chaperone for mitochondrial proteins [5], [6] and the activation of p53 [7]. Of particular interest, are the reports that prohibitin is required for the transcriptional activity of p53 [7], [8] since deregulation of p53 is a likely candidate that may contribute to the mammary gland carcinoma observed in MMTV-Skp2B mice [2]. Indeed, we also reported that Skp2B overexpressing cells show a defect in the transcriptional activity of p53 both *in vitro* and *in vivo* [4]. Further analysis of the tumors in MMTV-Skp2B mice revealed that they are characterized by the cleavage of insulin-like growth factor binding protein 4 (IGFBP-4) [2]. IGFBP-4 binds and titrates IGF-II away from the IGF receptor, therefore inhibiting IGF-II signaling [9], [10]. Whether the increased cleavage of IGFBP-4 [2] and the defect in p53 [4], we observed in MMTV-Skp2B mammary tumors, are related was however never addressed.

The defect in p53 activity was associated with an increase in the baseline levels of p53 protein in Skp2B overexpressing cells raising the possibility that the turn-over of p53 is reduced in these cells. The p53 core domain is intrinsically unstable. It is correctly folded at 37°C, but mild changes in temperatures have been reported to promote its spontaneous misfolding and denaturation [11]. Similarly, most mutations in p53 share the common property of reducing the thermostability, and cause p53 denaturation. Denatured p53 fails to promote the transcription of its ubiquitin ligase mdm2, resulting in the stabilization of denatured p53 and an elevation in its basal level. Chaperones such as Hsp90 and 70 assist the folding of p53 and prevents its denaturation [12]. Whether, a chaperone of p53 is affected in Skp2B overexpressing cells remain to be determined.

We therefore initiated this study to investigate the mechanism leading to the elevated proteolytic cleavage of IGFBP-4 and p53 levels in MMTV-Skp2B transgenic mice. We report two novel findings; 1) that the expression of the protease of IGFBP-4, PAPP-A, is repressed by wild type p53 but activated by denatured p53 and 2) that prohibitin acts as a chaperone of p53 such that upon Skp2B overexpression or treatment with siRNA against prohibitin, the conformation of p53 becomes denatured and mimics mutant p53. These results therefore offer a link between the increased cleavage of IGFBP-4 [2] and the defect in p53 activity [4] we had previously reported. Further, they suggest that hyperactivation of IGF-II signaling as a result of IGFBP-4 cleavage is likely to contribute to the oncogenic function of Skp2B.

## Results

### Skp2B overexpression and mutant p53 promote the transcription of PAPP-A

**Figure 1:**
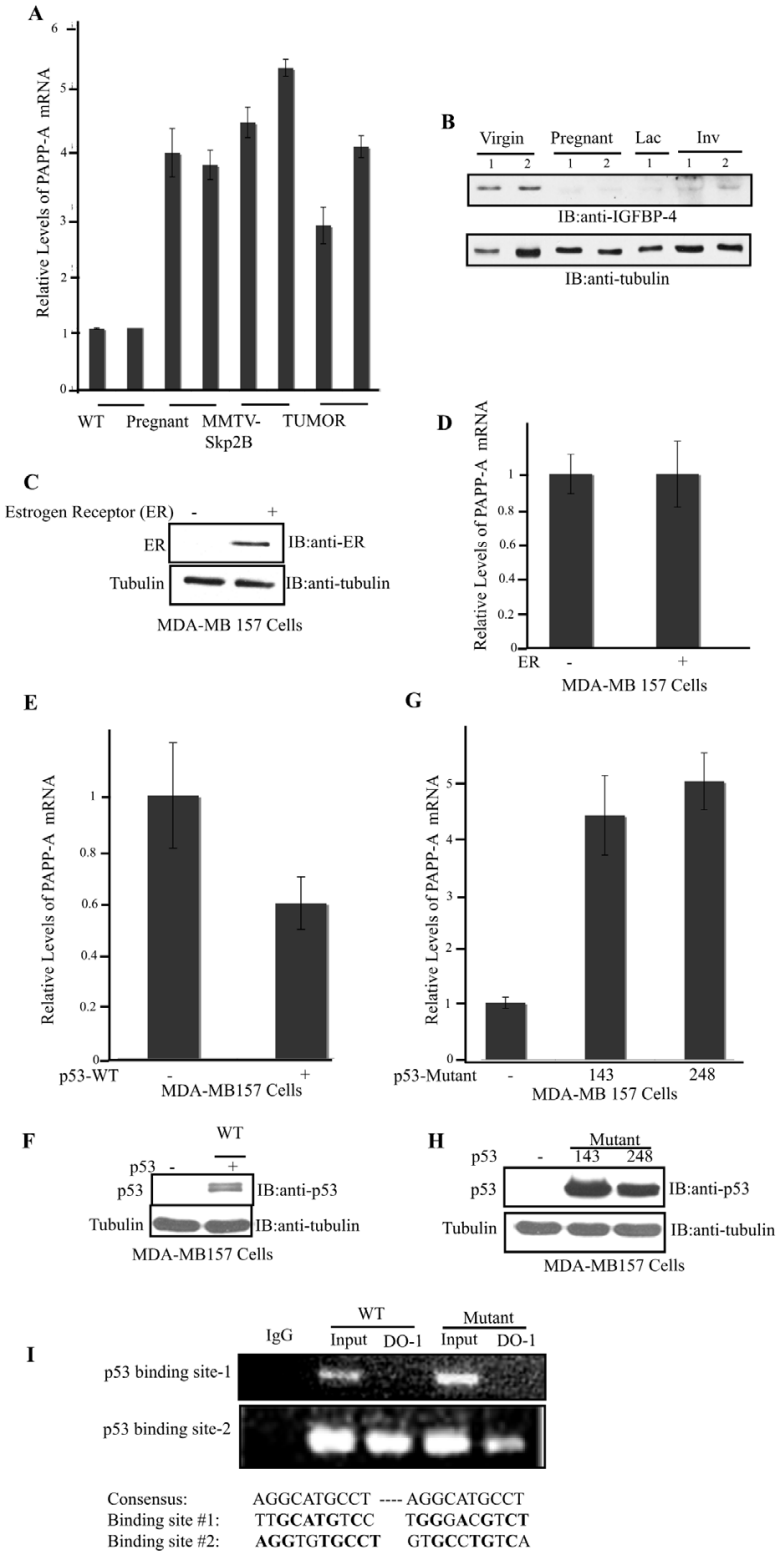
p53 regulates PAPP-A levels by binding to the PAPP-A promoter. A) The mammary glands from wild type, pregnant (wild type) MMTV-Skp2B and tumors were analyzed for the mRNA levels of PAPP-A by quantitative RT-PCR. B) The level of IGFBP-4 in the mammary gland of wild type mice that are either virgin, pregnant, lactating or involuting was determined by western blot. C) The estrogen receptor null breast cancer cells MDA-MB-157 were transfected with 3 ug of estrogen receptor plasmid and D) after 24 hours cells were harvested for analyzing the expression of the estrogen receptor and mRNA levels of PAPP-A by quantitative RT-PCR. E) The p53 null MDA-MB157 cells were transfected with 2 µg of wild type p53 and PAPP-A mRNA levels determined. F) The level of expression of transfected wild type p53 was determined by western blot. G) MDA-MB 157 cells were transfected with 2 µg of different mutants of p53 (p53–143 and p53–248), 24 hours after, cells were harvested for analysis of PAPP-A mRNA. H) Western blot showing the expression of mutant p53. Tubulin was used as a loading control. I) p53 binding site on PAPP-A promoter. MDA-MB157 cells transfected with 2 µg of wild type and mutant p53 constructs. After 24 hours of transfection cells were fixed with formaldehyde and collected for chromatin immunoprecipitation (ChIP) using the p53 (DO-1) antibody. Normal mouse IgG served as a negative control.

Pregnancy-associated plasma protein-A (PAPP-A) is the protease required for the cleavage and inactivation of IGFBP-4 [10]. Elevation in PAPP-A represents therefore a likely candidate for the elevation in IGFBP-4 cleavage we had observed [2]. As the antibodies against PAPP-A recognized human PAPP-A but not mouse PAPP-A, we used RT-PCR to determine the levels of PAPP-A in mice. We found that PAPP-A levels are 4–5 fold higher in the mammary gland of transgenic mice compare to non-transgenic mice (Fig. 1A). As a control, the levels of PAPP-A in the mammary gland during pregnancy in wild type mice were also determined. We found that PAPP-A levels increase during pregnancy suggesting that in addition of being secreted into the circulation from the placenta [13], PAPP-A is also specifically expressed in the mammary gland during pregnancy (Fig. 1A). To determine whether elevation in PAPP-A during pregnancy correlates with a decrease in IGFBP-4 during pregnancy, we next determine the levels of IGFBP-4 during pregnancy, lactation and involution of the mammary glands of wild type mice and found that its levels are low during pregnancy and lactation (Fig. 1B). These results show for the first time a correlation between PAPP-A expression and lost of IGFBP-4 in the mammary gland during pregnancy. Therefore, while PAPP-A expression in the mammary gland is restricted to pregnancy in wild type mice but is observed in virgin MMTV-Skp2B transgenic mice, Skp2B must promote the degradation of a substrate, which results in the abnormal expression of PAPP-A.

Since we found that Skp2B promotes the degradation of the repressor of the estrogen receptor activity (REA) [2] and of prohibitin [4], which leads to the repression of p53 transcriptional activity, we next tested whether the estrogen receptor (ER) or p53 could be responsible for the activation of PAPP-A transcription. For this experiment, the p53 and ER negative breast cancer cell line MDA-MB157 was used. First, MDA-MB157 cells were transfected with the ER and the level of PAPP-A compared using RT-PCR since PAPP-A is a secreted protease. We found no change in the level of PAPP-A following expression of the ER in these cells (Fig. 1C, D).

We next analyzed the effect of wild type and different mutants of p53 on the expression of PAPP-A. Upon transfection of wild type p53, the levels of PAPP-A mRNA were reduced by 2-fold (Fig. 1E, F). Conversely, transfection of two mutants of p53 (p53–143 and p53–248) in MDA-MB 157 cells resulted in an elevation of PAPP-A mRNA by 4–5 folds (Fig. 1G, H). These results indicate that while wild type p53 represses PAPP-A, mutant p53 gains the ability to activate the transcription of PAPP-A.

To further test the effect of p53 on the expression of PAPP-A, we analyzed the sequence of the PAPP-A gene for the presence of a p53-binding site. Since two putative binding sites were identified, we next tested their ability to bind p53 by ChIP. MDA-MB 157 cells were transfected with wild type and mutant p53 and after 24 hours, cells were fixed with formaldehyde, lysed, sonicated and ChIP assays were performed. We found that both wild type and mutant p53 bind to one of the p53 binding site but not the other (Fig. 1I). These results indicate that binding of mutant p53 to this site must either recruit activators or prevent binding of repressors to allow this site to act as an enhancer for the transcription of PAPP-A. Further, these results indicate that mutant p53 gains the ability to activate the transcription of PAPP-A independently of pregnancy.

### Elevated levels of PAPP-A mRNA in breast cancer cell lines expressing mutant p53

**Figure 2:**
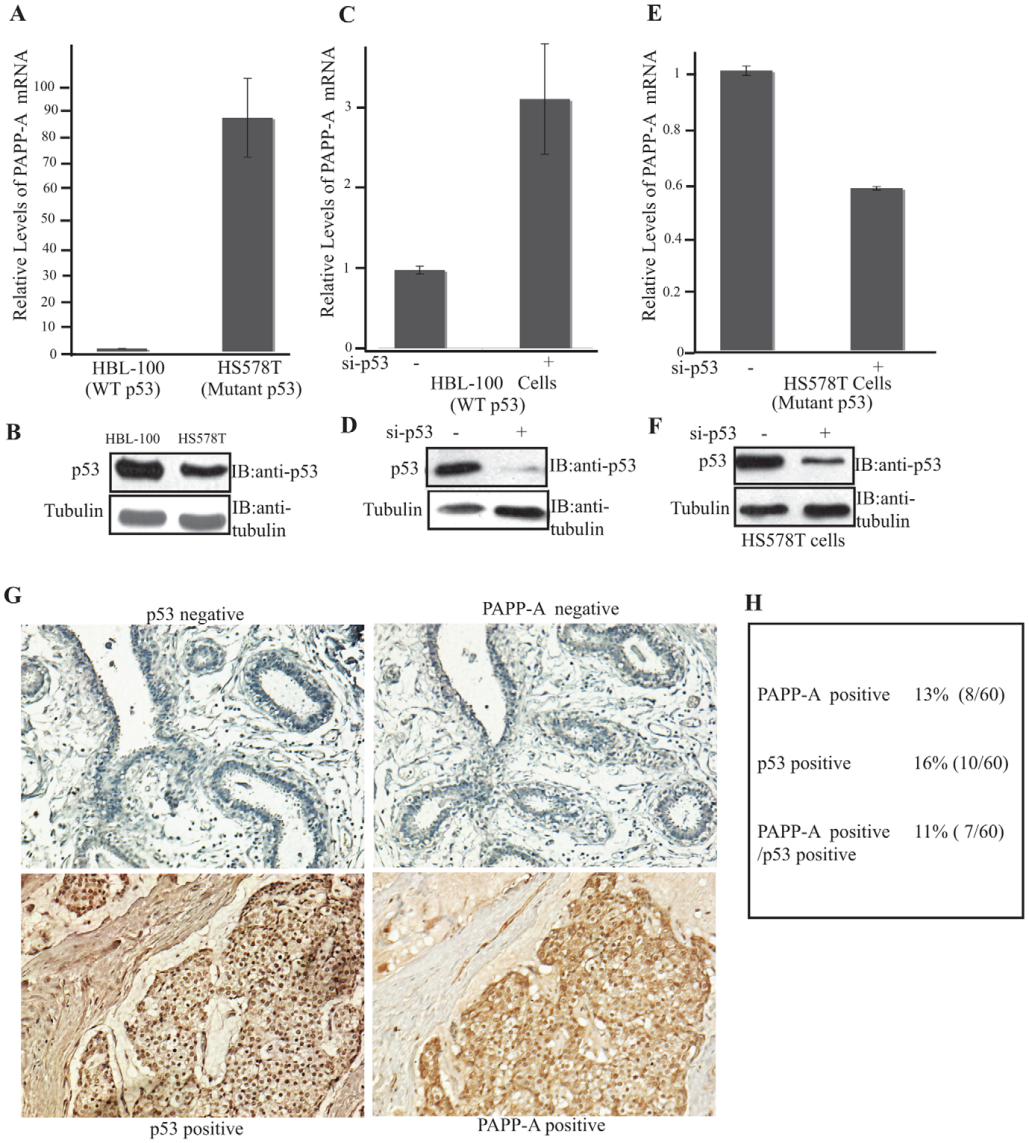
p53 regulates PAPP-A levels. A) HBL-100 cells express wild type p53, while HS578T cells express mutant p53. The levels of PAPP-A mRNA in these cell lines were determined by qRT-PCR. B) Western blot of the levels of p53 in HBL-100 and HS578T cells. C) the expression of p53 was inhibited in HBL-100 cells using siRNA and the PAPP-A mRNA levels were determined by qRT-PCR. D) the efficiency of siRNA against p53 was determined by western blot. E) the expression of p53 was inhibited in HS578T cells using siRNA and the PAPP-A mRNA levels were determined by qRT-PCR. F) the inhibition of p53 by siRNA in HS578T cells was monitored by western blot. G) Duplicate human breast carcinoma tissue microarray slides (Zymed) were stained by IHC using the DO1 antibody against p53 and the rabbit polyclonal antibody against PAPP-A (Dako). H) The percentage of tumors positive for p53, PAPP-A or both was determined.

To determine whether, a link exists between endogenous p53 and PAPP-A expression, total RNA was extracted from HBL-100 (wild type p53) and HS578T (mutant p53) cells. Total RNA was subjected to qRT-PCR using human specific primers for the detection of PAPP-A mRNA levels. We found that the level of PAPP-A mRNA was 87 fold higher in HS578T cells than HBL-100 cells (Fig. 2.A, B). Elevated levels of PAPP-A mRNA levels was also observed in other two breast cancer cell lines harboring mutant p53 (T47D and MDA-MB 231) (data not shown). To further explore the potential link between p53 and the expression of PAPP-A, we then tested the effect of inhibiting wild type p53 by siRNA on PAPP-A mRNA levels. HBL-100 cells were transfected with siRNA (30 µM) against p53 and PAPP-A mRNA levels were determined. We found that inhibition of wild type p53 led to a 3.2 fold increase in PAPP-A levels (Fig. 2.C, D) suggesting that wild type p53 represses the transcription of PAPP-A. Conversely, inhibition of mutant p53 siRNA (30 µM) in HS578T cells resulted in a reduction in PAPP-A mRNA levels (Fig. 2. E, F). This result supports the notion that mutant p53 activates the transcription of PAPP-A. Importantly, since PAPP-A is a secreted protein, elevation in the transcription of PAPP-A by p53 does not result in an elevation in the intracellular levels of PAPP-A protein. To circumvent this difficulty, we determine whether a correlation exists between mutant p53 and PAPP-A proteins using breast carcinoma tissue, which contains the stroma where PAPP-A resides following its secretion. Microarray duplicate slides were stained by immunohistochemistry (IHC) using either p53 or anti-PAPP-A antibody. Detection of p53 by IHC represents a stabilized form of p53 and therefore is interpreted as representing mutant p53. In our array, p53 staining was detected in 16% of samples (Fig. 2G, H), while PAPP-A was observed in 13% (Fig. 2G, H), further, of the 8 PAPP-A positive tumors, 7 were also positive for p53 (Fig. 2G, H). Therefore, these results support our vitro observations in human cell lines that mutant p53 promotes the transcription of PAPP-A.

### Skp2B overexpression promotes the accumulation of denatured p53

**Figure 3:**
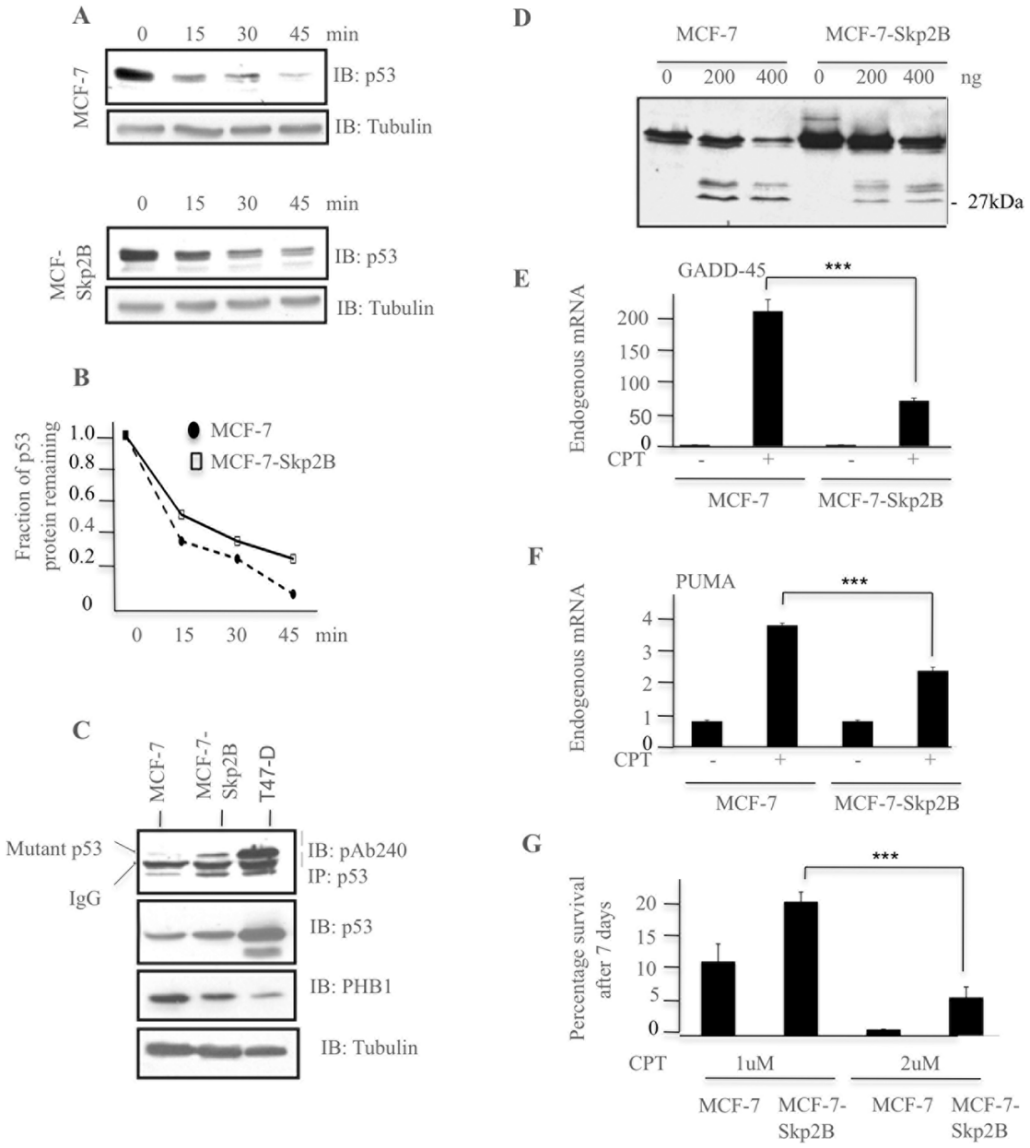
Skp2B overexpression promotes the accumulation of denatured p53 species. A) Cycloheximide chase in MCF-7 and MCF-7Skp2B cells was performed for the indicated time and cells were harvested for immunoblotting using anti-p53 antibody. Tubulin was used as a loading control. B) Quantification of the fraction of p53 remaining in MCF-7 and MCF-7Skp2B cells at the indicated time of cycloheximide treatment. C) Proteins from MCF-7, MCF-7Skp2B and T47D cells were used for immunoprecipitation of endogenous p53 using the mouse monoclonal pAb240 anti-p53 antibody, which immunoprecipitates only denatured but not wild-type forms of p53. The immunoblot was then developed using an anti-rabbit p53 antibody. D) Extracts from MCF-7 and MCF-7Skp2B were digested with increasing concentrations of thermolysin and the resulting cleavage products detected by immunoblot using the DO1 antibody. The 27kDa product is indicated. E) MCF-7 and MCF-7-Skp2B cells were treated with or without camptothecin (10 µM) for 4 hours and the levels of GADD45 mRNA levels determined qRT-PCR. The difference between the levels of GADD45 mRNA following DNA damage between MCF-7 and MCF7-Skp2B cells was statistically significant. The three arrows indicate a p value of less than 0.0001. F) MCF-7 and MCF-7-Skp2B cells were treated with or without camptothecin (10 µM) for 4 hours and the levels of PUMA mRNA levels determined qRT-PCR. The difference between the levels of PUMA mRNA following DNA damage between MCF-7 and MCF7-Skp2B cells was statistically significant. The three arrows indicate a p value of less than 0.0001. G) MCF-7 treated with mock siRNA and MCF-7-Skp2B cells were treated with 1 or 2 uM camptothecin for 7 days and the percentage of cells that survived determined. The difference between the percentage of cell surviving following DNA damage between MCF-7 and MCF7-Skp2B cells was statistically significant. The three arrows indicate a p value of less than 0.0001.

Since wild type p53 represses the expression of PAPP-A but both mutant p53 and Skp2B overexpression leads to activation of PAPP-A expression, we next aimed at determining the mechanism by which wild type p53 in Skp2B overexpressing cells mediates this effect. The first indication came from the observation that we consistently observed that the basal level of p53 is higher in Skp2B overexpressing cells compared to the parental cell line, while the activity of p53 is reduced [4]. To determine whether this observation may be due to a difference in the half-life of p53 between the two cell lines, we next performed a cycloheximide chase. We found that in MCF-7 cells, less than 10% of p53 protein was detected after 45 minutes (Fig. 3A, B), while in MCF-7Skp2B cells, 25–30% of p53 protein was detected at 45 minutes (Fig. 3A, B). This result indicates that the turn-over of p53 is reduced upon Skp2B overexpression and that p53 may be denatured in these cells.

While several different mutations exist, most mutations share the common property of reducing the thermostability, and cause p53 denaturation. This shared property of denatured p53 is reflected by the ability of the pAb240 antibody to recognize multiple different mutants since it detects denatured p53 [14]. Denatured p53 fails to promote the transcription of its ubiquitin ligase mdm2, resulting in the stabilization of denatured p53 and an elevation in its basal level. We therefore reasoned that one potential explanation for our observation is that in absence of prohibitin, due to Skp2B overexpression, a fraction of total p53 becomes denatured and therefore behaves like mutant p53. To test this possibility, we took advantage of the pAb240 anti-p53 antibody, which immunoprecipitates only denatured p53 but not wild-type p53 [14]. For this experiment, T47D breast cancer cells were used as a positive control since they express mutant p53. We found that as expected, in T47D cells, p53 levels were elevated (Fig. 3C, middle panel) due to a defect in mutant p53 turn-over and that mutant p53 was effectively immunoprecipitated in T47D cells by the pAb240 antibody (Fig. 3C, top panel). In contrast, pAb240 failed to immunoprecipitate p53 in MCF-7 cells since p53 is wild-type in these cells. In MCF-7Skp2B cells however, we found that a small but reproducible fraction of p53 was successfully immunoprecipitated by pAb240 (Fig. 3C). This observation raises the possibility that prohibitin prevents p53 denaturation such that in its absence, a fraction of p53 adopts a non-native conformation that resembles that of mutant p53, resulting in an increased stability.

The formation of denatured p53 in absence of prohibitin was further tested using the analysis of the pattern of cleavage products following thermolysin digestion. Thermolysin is a protease that has previously been shown to cleave p53 and generate among other products, a 27 kDa product containing the DNA binding domain [15]. Further, the pattern of cleavage products following thermolysin digestion between wild type p53 and mutants p53 was found to be different and also to vary between mutants [15]. We therefore compare the pattern of p53 cleavage products following thermolysin digestion in MCF-7 and MCF-7Skp2B cells. We found that despite the presence of more p53 in MCF-7Skp2B cells, the level of the 27 kDa product was lower than in MCF-7 cells (Fig. 3D). The ratio p53/27-cleavage product using 200 ng thermolysin was 3.3 in MCF-7 cells and 15.2 in MCF-7Skp2B cells indicating a 4.6 fold increase in the p53/27-product ratio.

Collectively, the observations that in MCF-7Skp2B cells; 1) the turn-over of p53 is extended, 2) pAb240 antibody precipitates p53 and 3) the pattern of p53 cleavage by thermolysin is different, all strongly argue that upon Skp2B overexpression a fraction of p53 adopts a denatured conformation. To further test the consequence of such altered conformation on the activity of p53, the levels of endogenous targets of p53 GADD45 and PUMA were analyzed following DNA damage using treatment with camptothecin. As we had reported for p21, a well described transcriptional target of p53 [4], the transcription of both of these endogenous targets were reduced in Skp2B overexpressing cells compared to the parental cell line (Fig. 3E, F).

We next tested the viability of MCF-7 or MCF-7Skp2B cells following treatment with 1 or 2 uM camptothecin. We found that while the viability of MCF-7 cells after 7 days of treatment with 1 or 2 uM camptothecin was 10% and 1% respectively, the survival was increased to 20% and 6% in MCF-7kp2B cells (Fig. 3G). Therefore, these results are consistent with a defect in p53 activity upon Skp2B overexpression.

### Prohibitin affects the folding of p53

**Figure 4:**
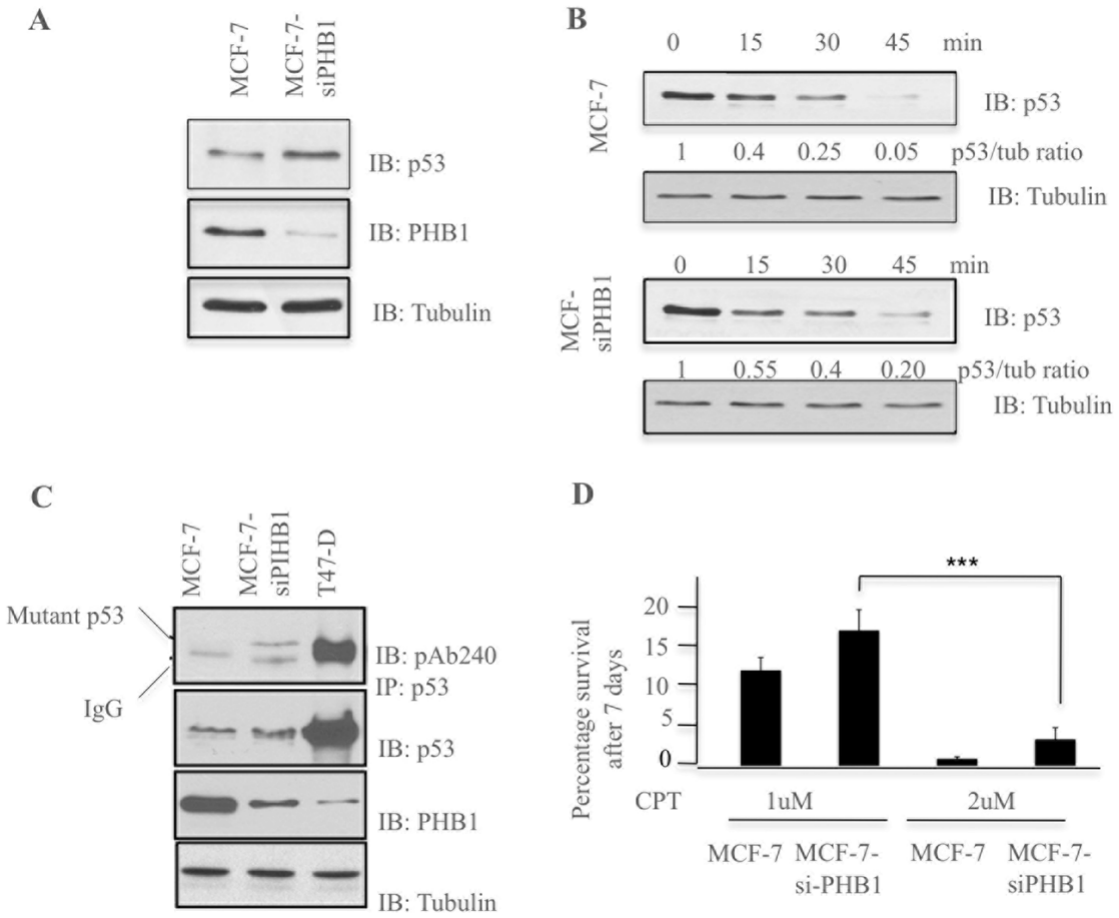
Prohibitin affects the conformation of p53. A) MCF-7 cells were transfected with siRNA against prohibitin for 48 hours. The inhibition of prohibitin expression was confirmed and the levels of p53 determined by immunoblot. B) Cycloheximide chase in MCF-7 and MCF-7 cells treated with siRNA against prohibitin was performed for the indicated time and cells were harvested for immunoblotting using anti-p53 antibody. Tubulin was used as a loading control. The quantification of the fraction of p53 remaining in MCF-7 and MCF-7Skp2B cells relative to the amount of p53 at time 0 is indicated at each time point of cycloheximide treatment. C) Proteins from MCF-7, MCF-7 transfected with siRNA against prohibitin and T47D cells were used for immunoprecipitation of endogenous p53 using the mouse monoclonal pAb240 anti-p53 antibody, which immunoprecipitates only mutant but not wild-type forms of p53. The immunoblot was then developed using an anti-rabbit p53 antibody. D) MCF-7 treated with mock siRNA and MCF-7 cells treated with siRNA against prohibitin were treated with 1 or 2 uM camptothecin for 7 days and the percentage of cells that survived determined. The difference between the percentage of cell surviving following DNA damage between MCF-7 and MCF7-siPHB cells was statistically significant. The three arrows indicate a p value of less than 0.0001.

Since Skp2B promotes the degradation of prohibitin [4] and that p53 is denatured in Skp2B overexpressing cells (Fig. 3), we then ask if prohibitin alone can affect the conformation of p53 using the same approaches. First, we inhibited the expression of prohibitin using siRNA in MCF-7 cells and determine the levels of p53 by western blot. We found that inhibition of prohibitin alone led to an increase in p53 levels (Fig. 4A). This elevation correlated with a decrease in the turn-over of p53 as determined by cyclohexmide chase (Fig. 4B). The p53/tubulin ratio at time zero was set at 1, and the p53/tubulin ratio was determined at each time point after addition of cycloheximide. This analysis revealed a p53/tubulin ratio of 0.05 and 0.20 after 45 minutes in MCF-7 and MCF-7siPHB1 respectively indicating that while 95% of p53 is degraded by 45 minutes in MCF-7 cells, in MCF7-siPHB1 cells, 20% of p53 remained at this time point (Fig. 4B). Further, the presence of denatured p53 in MCF-7 cells assessed using immunoprecipitation with the pAb240 antibody. As we had observed in Skp2B overexpressing cells (Fig. 3C), while no mutant p53 could be detected in MCF-7 cells, a fraction of p53 was detected in MCF-7 cells transfected when prohibitin is inhibited (Fig. 4C). The effect of inhibition of prohibitin on survival following treatment with camptothecin was also tested. We found that while 10% and 1% of MCF-7 cells survived after 7 days of 1 uM or 2 uM camptothecin, 17% and 4% survived in MCF-7Skp2B (Fig. 4D).

Collectively, these results indicate that as we have observed in Skp2B overexpressing cells, reducing prohibitin by siRNA leads to the denaturation of p53.

Taken together these results indicate that prohibitin plays an important role in the conformation of wild type p53 by preventing its spontaneous misfolding and denaturation.

## Discussion

**Figure 5:**
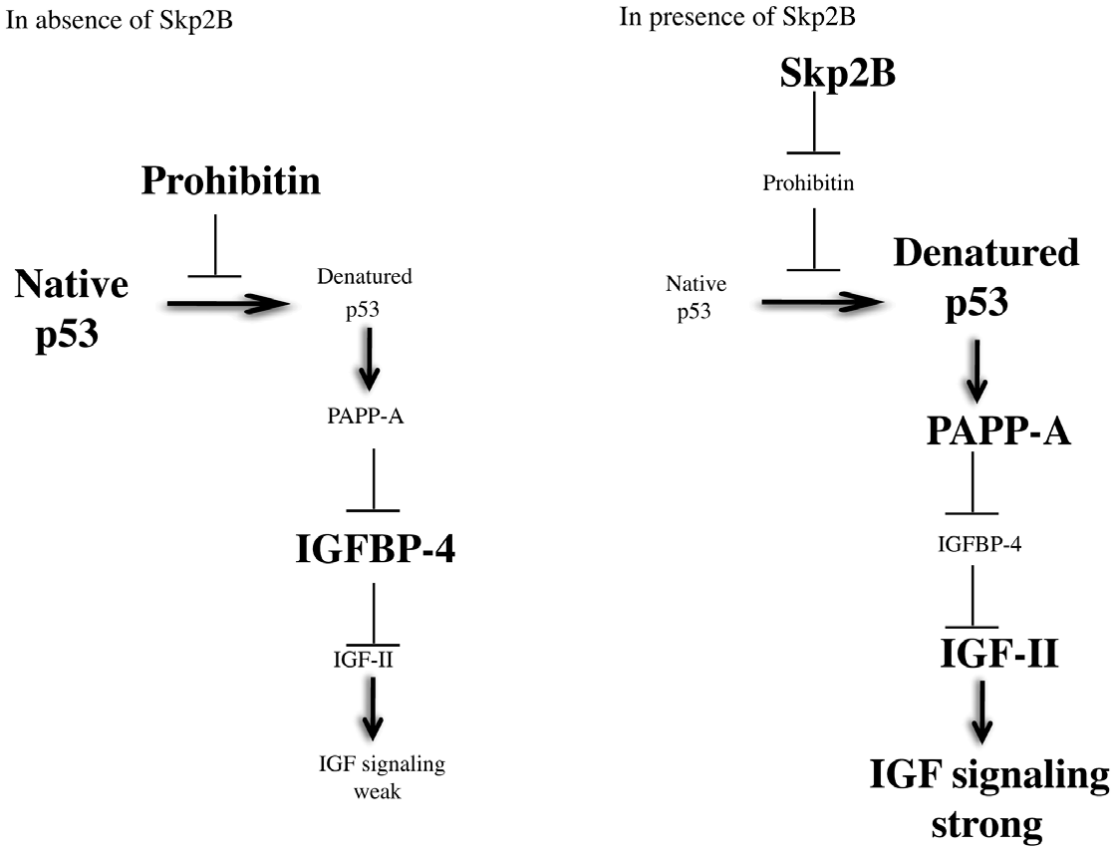
Model of the link between Skp2B-prohibitin-p53-PAPP-A–IGFBP-4 and IGF signaling.

We previously reported an attenuated transcriptional activity of p53 both in vitro and in vivo [4], as a result of the degradation of prohibitin by Skp2B. However, this inactivation of p53 was despite elevated levels of p53 in Skp2B overexpressing cells. We show here that this elevation in p53 levels is due a slower turn-over of p53 in these cells. Based on these observations, we propose a model (Fig. 5) whereby prohibitin acts as a chaperone and protects p53 from adopting a non-native conformation and become denatured. We postulate that while the majority of p53 adopts a native conformation (Large arrow Fig. 5), a small fraction may spontaneously engaged in an incorrect folding pathway. Our data support the hypothesis that prohibitin assists in preventing progression through this incorrect folding pathway, which otherwise lead to misfolding and denatured proteins that are more stable, such as mutant p53. When Skp2B is overexpressed, prohibitin is reduced such that the fraction of denatured p53 increases. In Skp2B overexpressing cells (Fig. 5), native p53 is rapidly degraded by Mdm2 and the denatured p53 fraction contributes to the elevated level of total p53 observed in these cells.

Also, we found that denatured p53 either due to a mutation of the loss of prohibitin gains the ability to transcribe PAPP-A (Fig. 5). Similarly to what has been reported for IGFR-1 [16], our data indicates that while wild type p53 represses the transcription of PAPP-A, mutant p53 activates its transcription. This result adds to the growing list of genes that are regulated by mutant p53 [17], [18], [19], [20], [21]. As for the mechanism by which mutant p53 exerts its effect on these genes, so far several different mechanisms have been reported, while the mechanism remain unclear for other genes.

Our result validates a p53 binding site in intron 1 of the PAPP-A gene suggesting that binding of mutant p53 to this site acts as an enhancer, rather than a direct effect of p53 on the promoter of PAPP-A. Transcriptional effect of p53 by binding to p53 site in intronic sequence is not novel as the transcription of GADD45, a well characterized transcriptional target of p53, is regulated by binding of p53 to a p53 binding site in intron 3 of the GADD45 gene [22], [23], [24]. Therefore, our finding expands this mode of action of p53.

Since PAPP-A inhibits IGFBP-4, our result also expands on the effect of p53 on the IGF signaling. Wild type p53 is known to repress the transcription of IGFR1 [16] and to induce the transcription of IGFBP-3, a negative regulator of IGF-I [25]. Both of these events contribute to the repression of IGF signaling. Our data indicates that in addition of these effects, by repressing the expression of PAPP-A, wild type p53 allows the levels of IGFBP-4 to remain elevated therefore inhibiting IGF-II (Fig. 5). Therefore, the collective effect of the repression of IGFR-1, activation of IGFBP-3 and repression of the protease of IGFBP-4 is the inhibition of the IGF system both at the level of the receptor and its ligands IGF-I and IGF-II. Conversely, mutant p53 activates the transcription of IGF1R [16], fails to activate the transcription of IGFBP-3 and as shown in the present study, activates the transcription of PAPP-A. This triple hit on the IGF pathway is likely to contribute to the observation that IGF signaling is activated in triple negative breast cancer where p53 is frequently mutated [26], [27].

In summary, our results identify a novel axis linking Skp2B-prohibitin-p53 and the activation of PAPP-A, which results in the increased cleavage of IGFBP-4 observed in MMTV-Skp2B transgenic mice. In light of the fact that PAPP-A appears to act as an oncogene [28], [29], [30], [31] and the potent role of IGF signaling in cancers [32], this novel axis further supports the oncogenic function of Skp2B.

## Materials and Methods

The authors declare that: All animal work has been conducted according to Mount Sinai School of Medicine IACUC Animal ethic committee and was approved by this committee (assurance number A3111-01).

### Cell lines, plasmids and transfections

HS578T and MDA-MB 231, MCF-7 and Skp2B overexpressing (MCF-7-Skp2B) cells were grown in Dulbecco's modified Eagle's medium (DMEM) supplemented with 10% fetal bovine serum. T47D, HBL-100 and MDA-MB157 cells were grown in RPMI 1640 supplemented with 10% fetal bovine serum. pCMV-neo-Bam empty vector and the vectors containing wild type (WT) p53 and mutant p53 (143, 248) were obtained from Derek Leroith. All cell lines were obtained commercially from ATCC (USA). All transfections of plasmid DNA were performed using the Mirus reagent (TransIT-LT-1, Mirus) according to the manufacturer's instructions.

### RNA Extraction and Real Time RT-PCR

Total RNA was extracted from cell lines and mammary tissues using RNeasy Plus Mini Kit (cat. No. 74134) and 100 ng of each sample was used in real-time RT-PCR reaction using Quantitect SYBR Green RT-PCR Kit (Cat. No. 204243) following the manufacturer protocols (Qiagen). Real-time PCR was performed and analyzed as described previously [1] [33]. The following primers for the quantification of PAPP-A (Human): Forward 5′-GTCAATGTTCCTTCCAGTGC-3′ Reverse 5′-CTTGTGCTTATTCTCTCGGGC-3′ and (Mouse): Forward 5′-GTTCGCCCCTGGTCGGCCATC-3′ Reverse 5′-CGGCCAGTTCTGGGCAGTCG-3′ were used in the study along with the beta-actin primers as loading control.

### Transfection of small interfering RNA (siRNAs)

siRNA transfections were performed using Hi-perfect Reagent (QIAGEN) according to the manufacturer's protocol. The following siRNAs were used in the study. siRNA for Luciferase was used a control: 5′-CUUACGCUGAGUACUUCCGATT-3′ and 5′-UCGAAGGUACUCAGCGUAAGTT-3′. For inhibiting p53: p53 ShortCut siRNA Mix (Cat# N2011S) was purchased from New England Biolabs and used at a concentration of 30 nM.

### Chromatin Immunoprecipitation (ChIp)

ChIP assays were performed using MDA-MB157 cells transfected with wild type p53 and mutant p53 (143) constructs. After 24 hours of transfection cells were used for chromatin immunoprecipitation, as described previously [34]. Cells were treated with 1% formaldehyde (Sigma) for cross-linking followed by lysis of cells in RIPA buffer and sonication. The resulted lysates were incubated with p53 antibody. DNA-p53 complexes were immunoprecipitated, using antibody against p53 (DO-1). DNA after reverse cross-linking and purification (Qia-quick PCR Purification Kit, Qiagen) was subjected to PCR using the primers (ChIp-qPCR assay GPH012853 (+)02A (for detecting binding site 1) and ChIP-qPCR assay GPH012853(+)10A (for detecting binding site 2), (SA Biosciences) for the amplification of p53 binding sites. PCR reactions were performed using 5 µL of the DNA from the immunoprecipitations or the 2 µL of DNA from the input as template. PCR cycling conditions were as follows: 94°C for 3 min; then 35 cycles of 94°C for 45 s, 58°C for 45 s, and 72°C for 45 s; followed by 10 min at 72°C as final extension.

### Western blotting and Immunoprecipitation

These methods were performed as described previously [1], and membranes (Perkin Elmer Life Sciences) were probed with the following antibodies: rabbit anti-Prohibitin (Biolegend), mouse prohibitin (Neomarkers) for immunoprecipitation, mouse anti-FLAG (Sigma), mouse anti-Skp2 (Zymed), mouse anti-tubulin antibody (Sigma), mouse anti-Myc antibody 9E10 to detect Myc-ubiquitin, p53 DO-1 (Santa Cruz), anti-IGFBP4 (Upstate), anti-estrogen receptor (Santa Cruz), anti-PAPP-A (Dako) and pAb240 (generous gift from J. Manfredi). Immunoblots were developed by ECL (Amersham Pharmacia Biotech).

### Luciferase assay

To measure the transcriptional activity of p53, MCF-7 and MCF-7-Skp2B cells were transfected with the reporter plasmids pGL3-E1bTATA or p21 promoter-pGL2. Cells were also transfected with a plasmid constitutively expressing the Renilla luciferase. After 24 hours, luciferase activity was measured using a dual luciferase reporter assay system (Promega) according to the manufacturer's instructions. To calculate the relative luciferase activities, the ratio of the respective firefly and Renilla luciferase activities were determined. Luciferase assays were quantitated using a TD-20/20 Luminometer.

### Proteolytic digests of p53

Thermolysin (Sigma) digests were carried out on 20 mg of total protein extracts from either MCF-7 or MCF-7Skpk2B cells at 20 C in 50 ml reaction buffer containing 25 mM Tris pH 8.5, 10 mM MgCl_2_ and 1 mM DTT and 200 or 400 ng thermolysin for 20 minutes as described previously [35].

#### Immunohistochemistry

For immunostaining of paraffin sections, a mouse monoclonal antibody against human p53 (Santa Cruz, DO-1) was used at a concentration of 1∶100 and the rabbit polyclonal antibody against PAPP-A (Dakocytomation) was used at a dilution of 1∶50. Immunohistochemical staining of PAPP-A and p53 were performed on archival formalin-fixed tissue sections (3 to 5 um), which were dewaxed and rehydrated in water. Antigen retrieval was achieved in a pressure cooker for 2 minutes in 10 mM sodium citrate pH 6.0. Endogenous peroxidase activity was blocked with 3% (v/v) hydrogen peroxide for 10 minutes. All buffer washes between antibody incubations were with 50 mM Tris-HCl pH 7.6. The primary antibodies were incubated for 1:30 hour at room temperature and detected with the LSAB+ kit peroxidase (DAKO, K0690), with the link biotinylated anti-rabbit or mouse IgG for 1 hour and streptavidin peroxidase for 15 minutes. Slides were developed with AEC (3-amino-9-ethyl-carbazole) chromogen (DAKO) for 15 minutes. Sections were then counterstained in haematoxylin and mounted with DPX.

#### Scoring assessment

Demonstration of p53 and PAPP-A staining was assessed according to both the extent and the intensity of the staining. The extent ranges on a scale from 0 to 5 where a score of 0 indicated less than 10% of the cells were stained, 1 for 11–25%, 2 for 26–50%, 3 for 51–75%, 4 for 76–90% and 5 for more then 90% of the cells. The intensity was scored on a scale from 0 to 3 where 0 is negative and 3 is the strongest intensity of staining. The score for extent and intensity was then added and considered weak if the total score was between 1–3, moderate between 4–6 and strong when above 7. In this study, cancer sections were considered positive for p53 and PAPP-a staining if they scored either moderate or strong.
